# Long-Term Cultured Human Term Placenta-Derived Mesenchymal Stem Cells of Maternal Origin Displays Plasticity

**DOI:** 10.1155/2012/174328

**Published:** 2012-03-26

**Authors:** Vikram Sabapathy, Saranya Ravi, Vivi Srivastava, Alok Srivastava, Sanjay Kumar

**Affiliations:** ^1^Center for Stem Cell Research, Christian Medical College, Bagayam, Vellore 632002, India; ^2^Department of Cytogenetics, Christian Medical College, Bagayam, Vellore 632002, India; ^3^Department of Hematology, Christian Medical College, Bagayam, Vellore 632002, India

## Abstract

Mesenchymal stem cells (MSCs) are an alluring therapeutic resource because of their plasticity, immunoregulatory capacity and ease of availability. Human BM-derived MSCs have limited proliferative capability, consequently, it is challenging to use in tissue engineering and regenerative medicine applications. Hence, placental MSCs of maternal origin, which is one of richest sources of MSCs were chosen to establish long-term culture from the cotyledons of full-term human placenta. Flow analysis established bonafied MSCs phenotypic characteristics, staining positively for CD29, CD73, CD90, CD105 and negatively for CD14, CD34, CD45 markers. Pluripotency of the cultured MSCs was assessed by in vitro differentiation towards not only intralineage cells like adipocytes, osteocytes, chondrocytes, and myotubules cells but also translineage differentiated towards pancreatic progenitor cells, neural cells, and retinal cells displaying plasticity. These cells did not significantly alter cell cycle or apoptosis pattern while maintaining the normal karyotype; they also have limited expression of MHC-II antigens and are Naive for stimulatory factors CD80 and CD 86. Further soft agar assays revealed that placental MSCs do not have the ability to form invasive colonies. Taking together all these characteristics into consideration, it indicates that placental MSCs could serve as good candidates for development and progress of stem-cell based therapeutics.

## 1. Introduction

The term Mesenchymal stem cells (MSCs) was coined by Caplan in 1991 [1]. MSCs are defined as the class of stem cells that has the potential to self-renew and differentiate into multiple cell lineages [2, 3]. The presence of mesenchymal stem cells in the bone marrow was hypothesized by Cohnheim in 1860s [4]. In 1920s, Maximow postulated the importance of the marrow stromal tissue in supporting the development and maintenance of blood and hematopoietic organs [5]. In 1960s, Friedenstein was the first to demonstrate stromal cells could be isolated from whole bone marrow aspirate based on differentiation adhesion to tissue culture plastic dishes [6]. In addition, MSCs secrete proangiogenic [7] and antiapoptotic cytokines and possess immunosuppressive properties [8]. Bone marrow MSCs are most commonly used and primary source of MSCs [9]. However, due to invasive nature of bone marrow aspiration and limited proliferative capacity, efforts are underway to identify abundant and reliable sources of MSCs for clinical applications [9]. Mesenchymal stem cells can be broadly grouped into two different subgroups adult MSCs and fetal MSCs. Adult MSCs are isolated from bone marrow, peripheral blood. Fetal MSCs are isolated from Placenta, amniotic fluid, umbilical cord and umbilical cord blood [10]. Placenta provides one of the most reliable and abundant source of MSCs [11]. Term placental tissues are discarded after birth, hence these tissues can be effectively utilized for research as well as clinical application without much ethical concern. In this paper, we systematically characterize the term placental MSCs isolated from cotyledons and validated that the isolated MSCs fulfill the genotypic and functional criteria laid out for a proper MSC [11, 12]. We have demonstrated that these MSCs have the ability to rapidly expand up to even 25–30 passages without compromising the chromosomal number, cell cycle or apoptosis pattern, phenotypic characteristics, pluripotency-associated endogenous gene expression profile, and differentiation capacity. Placental MSCs were able to transdifferentiate into other cell lineages thus exhibiting their inherent plasticity.

## 2. Materials and Methods

### 2.1. Collection of the Human Placenta Samples

The ethical committee of Christian Medical College (CMC), Vellore, approved the study. Following the written consent term placental samples were collected from donors after elective caesarean.

### 2.2. Cell Isolation

Term human placental MSCs were isolated from cotyledons present towards the maternal side of the placenta. The placental membrane from the maternal side of the placenta was cut open and about 80 g of cotyledons was exercised. The cotyledons was thoroughly washed with PBS and cut into small pieces. The blood clots present in the cotyledons were mechanically removed. The minced placental was once again washed with physiological saline and subjected to sequential digestion with trypsin and collagenase I. The tissues were incubated with 0.25% trypsin for 1 hour at 37°C. After trypsin digestion, the sample was filtered through 250 *μ*m metal sieve. The retentate was collected and subjected to second digestion with12.5 U/mL collagenase I for 1 hour at 37°C. Collagenase I digested tissue sample was passed first through 250 *μ*m metal sieve and filtrate collected was passed through 100 *μ*m cell strainer. The filtrate containing cell suspension after dual filtration stages were subjected to centrifugation at 300 g for 10 minutes. The cell pellet was resuspended in RBC lysis buffer and centrifuged at 300 g for 10 minutes. Finally, the cell pellet was resuspended in Mesenchymal expansion medium (*α*MEM + 10% FBS + 50 u/mL penicillin + 50 *μ*g/mL streptomycin + 1 mM L-glutamine) and plated into two 75 cm^2^ flasks.

### 2.3. Antibodies

Information on primary and secondary antibodies used for flow-cytometry and immunostaining experiments is provided in Supplementary Table 1 is available online at doi: 10.1155/2012/174328.

### 2.4. Flow Cytometry

Cells after trypsinization was equally aliquoted (1 × 10^5^ cells per reaction) into FACS tubes and stained on live cells with respective antibody. Unstained antibody and cells stained with isotype antibody acted as controls. Antibodies were added to the cells in dark to avoid bleaching. After addition of the antibody, the sample was incubated at room temperature in dark for 20 minutes. Cells were washed with 1 mL of DPBS without calcium and magnesium and centrifuged at 300 g for 5 min. The pelleted cells were resuspended in 300 **μ**L DPBS w/o calcium and magnesium and analyzed with a flow cytometer (FACS Calibur; Becton Dickinson). A minimum of 10^4^ gated events was acquired from each sample for analysis using cell quest.

### 2.5. Cytogenetic Analysis

Karyotyping of human placental MSCs was carried at Passages 5 and 25 to verify the chromosomal integrity. Metaphase chromosomal preparations were performed according to standard procedures at a 400–550 GTG band level. Zeiss axioplan microscope was used to identify and analyse the chromosomes. Images were analyzed with a photometrics charged coupled device camera and controlled with smart capture imaging software.

### 2.6. Immunostaining

The cells cultured in 6-well plates were blocked with PBS (without Ca^2+^ and Mg^2+^) containing 0.1% BSA, fixed with 4% paraformaldehyde and permeabilized using 0.2% Triton X-100. If using unconjugated antibody, samples were first incubated with primary antibody, blocked with PBS containing 0.1% BSA and subsequently incubated with fluorescent dye conjugated secondary antibody. All cell samples were additionally counterstained with Hoechst 33342. Images were taken using leica DMI6000B (Leica) equipped with DFC360FX digital camera and analyzed with Lecia AF imaging software (Leica).

### 2.7. Total RNA Isolation and Reverse Transcription Polymerase Chain Reaction (RT-PCR)

Total RNA isolation was carried out using Trizol (Invitrogen). cDNA was prepared with superscript III reverse transcriptase enzyme. The primer sequences and their respective annealing temperature are presented in supplementary entary Table 2. PCR conditions were initial denaturation at 94°C for 2 min, followed by denaturation at 94°C for 1 min, annealing for 1 min, extension at 72°C for 2 min for 35 cycles, and final extension was carried out at 72°C for 5 min. Glyceraldehyde 3 phosphate dehydrogenase (GAPDH) RNA was used as a control for normalization of RNAs. PCR products were analyzed using ethidium bromide stained 2% agarose gels. Analysis of the gel images was carried out (Supplementary Table 2).

### 2.8. QPCR

Total RNA was extracted with Trizol (Invitrogen) according to the manufacturer's protocol. cDNA synthesis was carried out using Superscript III First-Strand synthesis system (Invitrogen). qRT-PCRs were carried out with SYBR Green master mix and AB real-time thermocycler (AB 7500). Primer sequences for the analysis of endogenous pluripotency gene expression are mentioned in the table below. The expression levels of individual genes were normalized against *β*-Actin (Supplementary Table 2).

### 2.9. Cell Cycle Analysis

For cell cycle analysis [13], cells were fixed with cold methanol, treated with RNase A 10 *μ*g/mL, stained with Propidium Iodide 50 *μ*g/mL, and analyzed by flowcytometer.

### 2.10. Apoptosis Analysis

Apoptosis analysis was carried by following the manufacturer's instructions (BD Pharmingen Annexin V). The cells were subjected to live staining with Annexin V and 7-AAD and analyzed the cells through flowcytometer.

### 2.11. Oligo-Lineage Differentiation Analysis

Placental MSCs at various passages were subjected intra- and translineage differentiation protocol to analyze the plasticity of the cells. After differentiation, cells were stained with appropriate stains and examined microscopically under Leica microscope.

### 2.12. Adipogenic Differentiation

Placental MSCs at 5 × 10^4^ cells were seeded onto 24-well plate (corning) containing adipogenic differentiation medium (Invitrogen) for 30 days, fresh medium added every 48 hours. Oil red O staining was carried out to visualize the presence of fat droplets. Cells were fixed with 4% paraformaldehyde, washed with sterile water, and incubated with 60% isopropanol at room temperature. Fixed cells were stained with 0.5% oil red O in isopropanol for 20 minutes at room temperature. After staining, cells were first washed with 60% isopropanol later rinsed with sterile water before observing under the microscope for imaging.

### 2.13. Chondrogenic Differentiation

Chondrogenic differentiation was carried out using falcon25 static cell culture system (specially fabricated in our lab for chondrocyte differentiation). Cells were subjected to micromass cell culture conditions to induce the chondrocyte differentiation under chondrocyte differentiation medium (Invitrogen) for 30 days. One million MSCs were pelleted at 300 g and chondrocyte differentiation medium was added without disturbing the pellet. Media was changed every 48 hrs. After differentiation, cells were fixed with 10% formalin, stained with merchrome, and embedded in paraffin. Staining on deparaffinized 5 *μ*m sections staining for proteoglycans was carried out using saffranin O and 3% alcian blue. After staining, sections were rinsed with distilled water, air dried at room temperature, immersed in xylene, and mounted using DPX before observing under microscopy.

### 2.14. Osteogenic Differentiation

For osteogenic differentiation, 5 × 10^4^ cells were seeded per well in 24-well plate containing osteogenic induction medium (Invitrogen) for 30 days, with media change every 48 hrs. After differentiation, presence of extracellular calcium was confirmed by VonKossa staining. For vonkossa staining, the cells were fixed in pre-cooled methanol. After fixing, the cells were washed with DPBS (W/O Ca^2+^ and Mg^2+^), treated with 5% silver nitrate solution in water, and exposed to UV light for 1 hour under the laminar hood. Stained cells were washed with water and incubated with 5% sodium thiosulphate in water for 2 min at room temperature. Finally, sample was rinsed with sterile water and observed under the microscope for imaging.

### 2.15. Myotubule Differentiation

For myotubule differentiation [14], 5 × 10^4^ placental MSCs were seeded in 25 cm^2^ flask containing mesenchymal expansion medium with 3 *μ*M 5-azacytidine. The cells were cultured for 21 days with media changes every 7 days. The cells were stained with Hoechst 33342 (5 *μ*g/mL), incubated at 37°C for 30 minutes before observing under the microscope for imaging.

### 2.16. Tubular Assay

Matrigel (BD) was thawed at 4°C for overnight. 50 *μ*L of matrigel was aliquoted per well of 96 well plate using precooled tips. The plate was centrifuged at 300 g for 5 min, 4°C. Allowed to polymerize at 37°C for 30 min. MSCs at 1 × 10^5^ cells/well were seeded in mesenchymal expansion medium. Cells were incubated at 37°C under hypoxic condition for 6 hours before observing under the microscope for imaging [15].

### 2.17. Neural Differentiation

To induced neuronal differentiation [16], 5 × 10^5^ placental MSCs were seeded onto serum-free *α*-MEM containing 5 mM *β*-mercaptoethanol and cultured for 6–9 hrs. The cells after induction were fixed for immunostaining analysis.

### 2.18. Retinal Cell Differentiation

For Retinal differentiation [17], 1 × 10^5^ cells were seeded into media containing Mesenchymal expansion medium supplemented with 50 *μ*M Taurine with 1 mM Beta-mercaptoethanol. The cells, were cultured for 4 days with media changes every 4 days. After retinal induction cells were collected in trizol for RT-PCR analysis or fixed for immunostaining.

### 2.19. Pancreatic Progenitor Cell Differentiation

For pancreatic differentiation [12, 18, 19], 25 cm^2^ flasks were treated with gelatin and 5 × 10^5^ cells were seeded onto gelatinized dish containing mesenchymal expansion medium with 10 mM nicotinamide and 1 mM *β*-Mercaptoethanol for 24 hrs. Following preinduction, cells were treated with Mesenchymal expansion medium without FBS but containing 10 mM nicotinamide and 1 mM *β*-Mercaptoethanol for 6 hours, and for following 18 hrs cells were treated with induction media containing FBS. After differentiation, cells were collected in trizol and subjected to RT-PCR analysis or fixed for immunostaining.

### 2.20. Soft Agar Assay

For Soft agar assay [20], 0.6% agar containing MEM was layered on the surface of 35 mm dish (corning) and incubated in laminar hood for 30 min. Later, 2 × 10^4^ MSCs were mixed with 0.3% agar containing MEM and overlayed on the top of 0.6% agar layer. Plate was incubated in hood for 20 minutes. Following incubation, 500 *μ*L of Mesenchymal expansion medium was added and incubated for 21 days. To the dish, 500 *μ*L of fresh media was added every 7 days once. HeLa cells were used as a positive control. 

### 2.21. Dithizone Staining

For Dithizone (STZ) Staining, the cells were incubated with DTZ solution 100 *μ*g/mL in *α*-MEM media for 20 minutes at 37°C. After staining, the cells were rinsed with twice with PBS and examined under microscope [21].

### 2.22. Cell Population Doubling Time (Gt)

Population doubling time indicates the growth rate of the placental MSCs [22], population doubling (PD)

(1) $$\text{PD}=\frac{\ln\left(\text{Nf}/\text{Ni}\right)}{\ln2},\;$$

where ln equals natural logarithm, Nf equals final cell count, Ni equals initial cell count

(2) $$\text{Gt}\;=\frac{t}{\text{PD}},$$

*t* = Time in hours after cell seeding.

Average Gt value was attained by adding the obtained Gt values for different experiments divided by number of experiments.

## 3. Results

### 3.1. Derivation of Adherent Fibroblast Like Mesenchymal Stem Cells (MSCs) from Maternal Side of Human Placenta and Immunophenotypic Characterization of Human Placental MSCs

**Figure 1:**
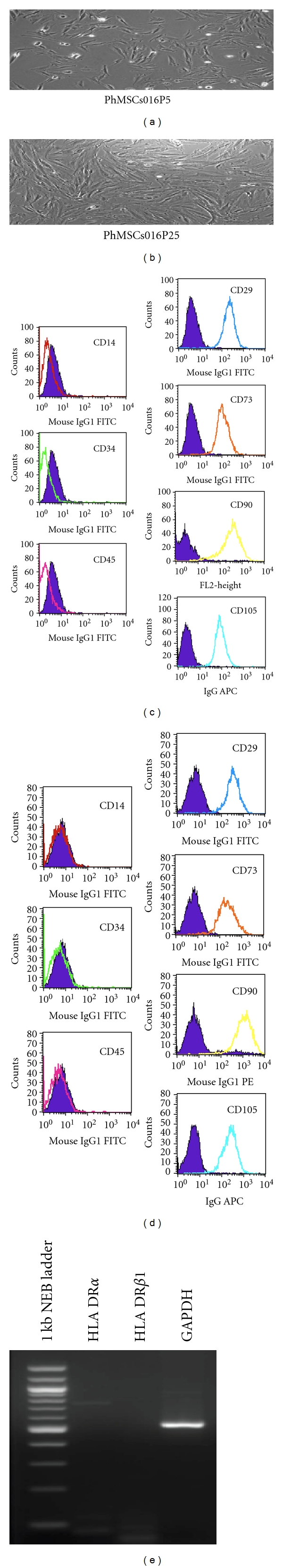
Morphology and characteristics of placental MSCs. (a) morphology of the placental MSCs at passage 5; (b) morphology of the placental MSCs at passage 25; (c) flowcytometric analysis of Placental MSCs at passage 5; (d) flowcytometric analysis of Placental MSCs at passage 25; (e) RT-PCR analysis of placental MSCs (PhMSCs 020P3) for MHC class II antigens.

Enzyme-mediated fractionation of human termed placenta resulted in derivation of fibroblast-like cells, which are generally term placenta-derived multipotent mesenchymal stem cells (PD-MSCs). Selection for MSCs rested on the classic adhesion method on tissue culture plastic. Placental MSCs from 8 term placental samples have been established from maternal side lobules of human placenta following trypsin digestion and collagenase-I treatment following which samples were passed through the 100 *μ* filter and were seeded in *α*-MEM containing 10% FBS, and adherent cell population was then characterized for their proliferation capabilities, cell cycle, apoptosis pattern, immunophenotypic features, and differentiation capabilities. The isolated MSCs formed a homogenous monolayer of adherent spindle-shaped fibroblast-like cells. The protocol proved successful in 8 of 8 placental tissues collection. Plating of cell suspensions from the first digest with trypsin did not produce any colonies, but cell suspensions produced from final collagenase I digest of placental tissue fragments typically produced MSC colonies of variable sizes that contained outgrowing fibroblast-like cells. After initial plating of the cells, the colonies became visible after 7 days. These MSC colonies in turn started to proliferate steadily, the flask was almost 60–70% confluent and ready for splitting by day 14. Typically, approximately 5–6 × 10^4^ cells were obtained within 12–14 days after plating. Following the process of initiation the flasks were subjected to trypsinization in 1 : 2 or 1 : 3 ratio. The 75 cm^2^ at 1 : 2 splitting was subconfluent by day 3, indicating these isolated cells had very rapid proliferating capacity. Outgrowing cells when harvested and replated in higher dilutions rapidly formed secondary colonies from single cells (Figures 1(a) and 1(b)). PD-MSCs were expandable up to passage 25–30 (as far as we cultured) without any changes in the morphological characteristics (Figures 1(c) and 1(d)) and were amenable to routine cryopreservation, thawing and differentiation protocols. The MSCs were characterized using flow-cytometry-based positive reaction for mesenchymal lineage surface markers CD29^+^, CD73^+^, CD90^+^, CD105^+^; and negative for hematopoietic marker CD34^−^, CD45^−^, also negative expression of CD14^−^, HLA DR^−^; was used to define MSCs (Figures 1(c), 1(d), and 1(e)). Flow cytometry revealed very little scatter in the phenotypic marker profile of placenta-derived isolates between all 8 cases, also population doubling time calculated were not significantly altered. The expression profile confirmed to the criteria generally defined for multipotent mesenchymal stem cells [23].

### 3.2. Plasticity of MSCs

**Figure 2:**
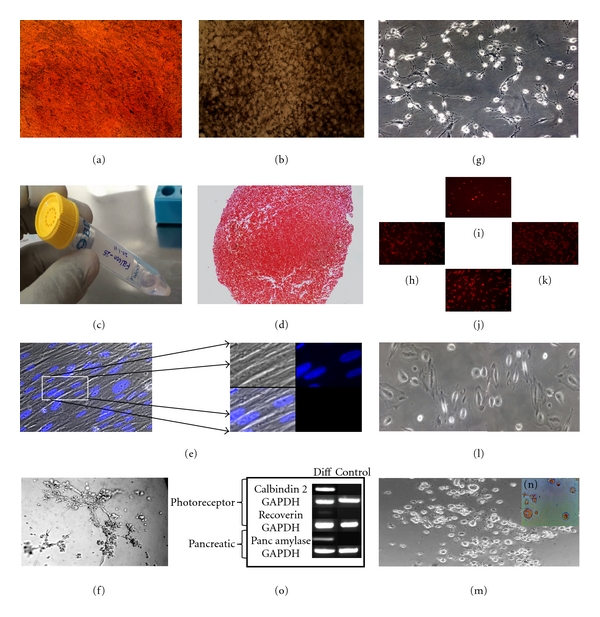
Pluripotency property of placental MSCs. (a) Oil red O staining (PhMSCsP5); (b) Von Kossa staining (PhMSCsP10); (c) “Falcon 25” static micromass cell culture system for chondrocyte differentiation; (d) saffranin O staining (PhMSCsP5); (e) hoechst 33342 staining of myotubules; (f) tubular assay; (g) neural differentiation of placental MSCs (PhMSCsP20); (h) map2 staining (PhMSCs021P15); (i) NeuN staining (PhMSCsP15); (j) GFAP staining (PhMSCs021P15); (k) Neural filament staining (PhMSCsP15); (l) Retinal cell differentiation of placental MSCs (PhMSCsP9); (m) Pancreatic progenitor cell differentiation of placental MSCs (PhMSCsP9) (n) dithizone (DTZ) positive pancreatic progenitor cells; (o) PCR analysis of ectodermal lineage (photoreceptor genes calbindin2 and recoverin) and endodermal lineage (pancreatic amylase gene).

Specific induction of differentiation was investigated with PD-MSCs, one early, one mid, and one late passage from all 8 subjects. This confirmed that the mesenchymal stemness profile by PD-MSC populations indeed associated with the ability to generate different mesodermal lineage cell types on their exposure to soluble growth and differentiation factors *in vitro*. At the same time, when subjected to translineage differentiation MSC shows remarkable plasticity to differentiate into ectodermal (neuronal cells, retinal cells) and endodermal lineage (pancreatic beta cells). Subconfluent culture was found critically important to maintain the stemness phenotype of PD-MSCs during expansion. The phenotypic profile of PD-MSCs when subcultured at 50–70% cell density remained unaffected, also maintained their initial marker profile and their ability to differentiate as well. MSCs can be differentiated into cells from all the three germ layers under suitable supplementary conditions *in vitro*. The figures display representative results of adipogenic (Figure 2 (a)), osteogenic (Figure 2 (b)), and chondrogenic (Figures 2 (c), and 2 (d)) differentiation assays, visualizing large lipid vacuoles, mineralized bone with calcium deposits and saffranin O positive collagen matrix respectively. These adipogenesis, osteogenesis, and chondrogenesis along with myotubule formation (Figure 2 (e)) and endothelial cells tubular assay (Figure 2 (f)) indicates the ability of the MSC to differentiate into mesodermal cell lineage. Moreover, reports are available on MSC culture in presence of the angiogenic growth factor VEGF induced expression of CD34, which is a marker of hematopoietic, as well as endothelial, precursors [35]. Figure also shows neurogenesis (Figures 2 (g), 2 (h), 2 (i), 2 (j), and 2 (k)) and retinal cell (Figure 2 (l)) differentiation which exhibits the ectodermal differentiation capacity of MSCs. Further, differentiation in pancreatic beta cells indicates (Figure 2 (m)) the endodermal differentiation capacity of placental MSCs. Also, RT-PCR amplification of calbindin2 and recoverin genes shows (Figure 2 (n)) retinal (ectodermal lineage) differentiation, and pancreatic amylase gene (Figure 2 (n)) was also amplified after pancreatic beta cell induction.

### 3.3. Extensively Passaged Placenta-Derived MSC Does Not Significantly Alter the Cell Cycle or Apoptotic Pattern While Maintaining the Normal Karyotype

**Figure 3:**
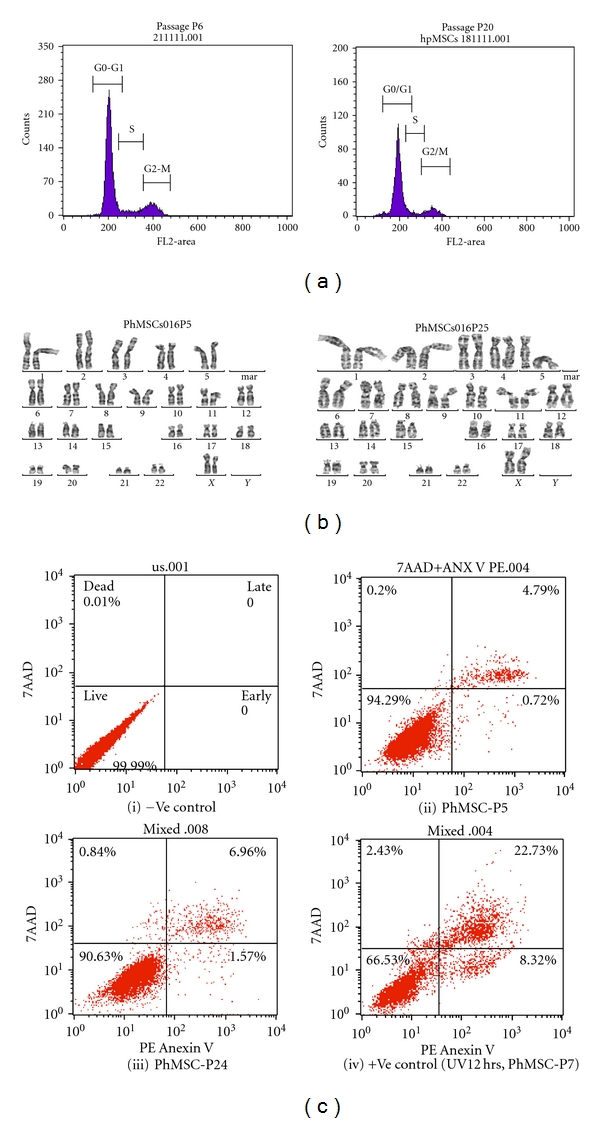
Cell cycle karyotype analysis: (a) cell cycle pattern of early (passage 5) and late passage (passage 20). PD-MSC were analyzed by FACS after propidim iodide staining. (b) Karyotype analysis was performed on early (passage 5) and late (passage25) passage MSC. (c) Apoptosis analysis was done by FACS using Annexin V and 7AAD. (i). negative control. (ii) Total % apoptotic MSC cells (Passage 5). (iii) % apoptotic cells (Passage 24) (iv).positive control.

In the next set of experiments after propidium iodide staining, we tested MSC cell cycle status; Figure 3(a) shows during early and late passaging there was not significant change in the cell cycling process. As detailed in Figure 3(b), karyotypes were normal 46, XX in all test samples. Chromosome number was found normal in all analyzed PD-MSC isolates (*n* = 8). Looking at maternal origin, we found that PD-MSC isolates obtained with our isolation procedure were always of maternal origin. Also, it was important to document the apoptosis pattern of the each passage proliferating MSC; Annexin-V and 7AAD stainning did not show (Figure 3(c)) significant change in the percentage apoptotic cells (~5–7% cells).

### 3.4. Placental MSCs Displays Higher Endogenous Gene Expression of Oct4, Sox2 and Nanog Compared to BM-Derived MSC

**Figure 4:**
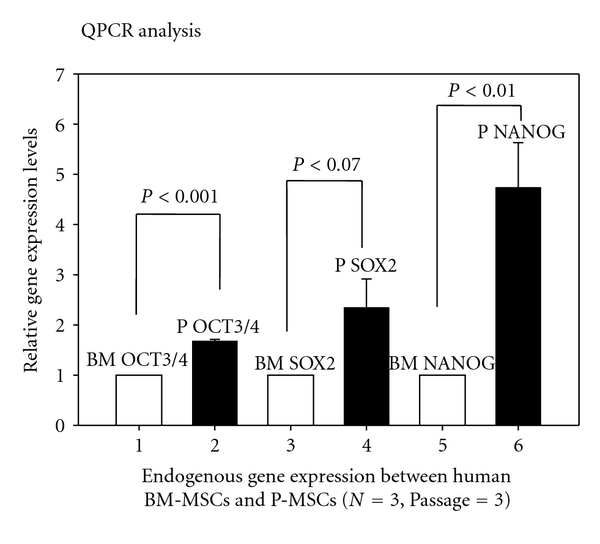
Comparative analysis of pluripotency associated endogenous gene expression between human marrow derived MSC and human placenta-derived MSC. Oct4. Sox2, and nanog gene expression profiles of bone-marrow-derived MSC and PDMSC analyzed by real-time qPCR analysis; error bars represent SE in five separate experiments.

FACS analysis by Oct3/4, Stro-1 antibodies did show positive reaction. Next, we wanted to analyze the pluripotency-associated endogenous gene expression profiles of PD MSCs and bone-marrow-derived MSC (BM-MSC).  Figure 4 shows data from comparative real-time qPCR, which revealed higher expression levels of Oct4, Sox2, and nanog compared to BM-MSC. Reports are also available of flow cytometry and immunocytochemistry, which revealed that PD-MSCs were positive for stage-specific embryonic antigen SSEA-3 but negative for SSEA-4 [11].

### 3.5. In Vitro Tumor-Genesis Detection Assay

**Figure 5:**
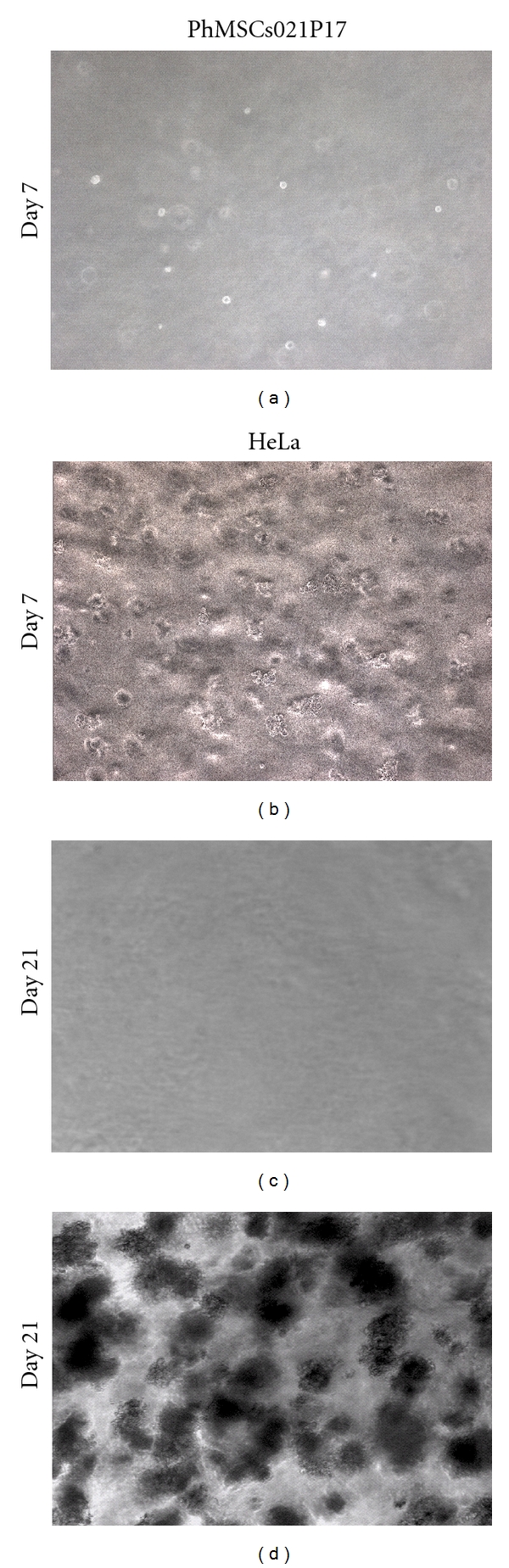
Soft agar assay. (a)Placental MSCs day 7; (b) HeLa cells day 7; (c) Placental MSCs day 21; (d) HeLa cells ay21.

Placental MSCs when subjected to soft agar assay did not yield tumoroids even after 4 weeks of in vitro culture in soft agar assay (Figure 5). However, HeLa cells began to form aggregates within 7 days, and many bigger colonies were formed at the end of day 21 (Figure 5).

## 4. Discussion

The human embryonic stem cells (ESCs) have the potential to differentiate into all the three cell lineages [24]. However, some of the practical and ethical concerns render them in usable for day-to-day clinical applications. Nonetheless, extra embryonic tissues can be effectively used to isolate pluripotent stem cells. Placenta is one of the extra embryonic organs that has rich source of progenitor or stem cells [25]. Placenta has two sides; one is foetal side consisting of amnion and chorion and other is the maternal side consisting of deciduas [24]. Mesenchymal stem cells (MSCs) isolated from maternal side of human term-placenta represent an important cell type for stem cell research and clinical therapy not only because of their ability to differentiate into mesodermal lineage cells, such as osteocytes, chondrocytes, muscle, or endothelial cells [2], but also for their remarkable translineage differentiation capabilities like neuronal cells, retinal cells (ectodermal), and pancreatic beta cells (endodermal lineage). In addition, they secrete large amounts of proangiogenic and antiapoptotic cytokines [26] and possess remarkable immunosuppressive properties [27]. MSCs have been derived from many different organs and tissues [28]. Evidence has emerged that different parts of human placenta, umbilical cords and amniotic membrane, as well as umbilical cord blood harbor MSC [29–32]. These tissues are normally discarded after birth, avoiding ethical concerns [23] Mechanical, as well as enzymatic, methods for MSC isolation from different regions of human placenta of different gestational ages have been reported in literature [29, 33–47]. Knowledge about vitality, average population doubling time, stable karyotype, cell cycle and apoptosis pattern, phenotype, and expandability of such placenta-derived MSC isolates is a prerequisite for therapeutic application; however, systematic investigations into reliability of this MSC source and phenotypic stability did not get that much attention. Furthermore, reports on placenta-derived MSCs often lack information about the cell cycle, apoptosis pattern, progenitor-specific endogenous gene-expression profile, and karyotype of the cell isolates. In this paper, we describe enzymatic fractionation of term-human placenta that facilitates recovery of oligo-lineage, fibroblast-like cells, which generally are termed as placenta-derived mesenchymal stem cells (PDMSCs) with high fidelity. As demonstrated by cell cycle or apoptosis analysis of cells from early as well as late passages; with average unaltered population doubling time, PD-MSC did not shows significant variations in either cell cycle or apoptosis pattern. Also, genotypic analyses of cell isolates from most of placental tissues were of maternal, not fetal, origin. Our systematic characterization of cell isolates from multiple cases showed that these cell isolates reproducibly fulfill the general definition of MSCs by both phenotypic and differentiation capabilities criteria. [24]. We demonstrate that maternally derived PD-MSCs can be greatly expanded, do not alter significantly change their cell cycle or apoptosis pattern, show pluripotency-associated endogenous gene expression, and maintain their differentiation capacity and stable phenotype displaying unaltered kayotype up to passage 25–30 passages. In these experiments, the placental MSCs were isolated from the cotyledons present in the maternal side of the placenta. Our method of cell isolation by way of sequential digestion of the trophoblast cell layer with trypsin and following digestion of remaining placental tissue with collagenase I proved very effective for obtaining PD MSCs. Outgrowth of PD-MSCs from collagenase I digests was successful in 8 of 8 placental tissues and resulted in populations with remarkably little scatter in their MSC profiles, between subjects. As for propagation, we found out that PD-MSCs must be propagated in subconfluent culture to maintain their MSC profile, because confluent culture led to gradual loss of MSC identity. With proper subconfluent passage, PDMSCs maintained their phenotypic MSC profile up to 30 passages. The flow cytometry studies indicate there is significant similarity in surface marker characteristics from passage 1 till passage 30. Microscopic observations revealed that placental MSCs proliferate rapidly till passage 30 without compromising on the morphological features and quality of the mesenchymal stem cell properties like cell cycle and apoptosis pattern, pluripotency-associated endogenous gene expression, and normal karyotype.

The characteristic data beyond passage 30 has not been tested in this study. The MSCs had spindle shaped fibroblast morphology. The absence of HLA DR*α* and HLA DR*β*1 expression, analyzed from RT-PCR, results indicate that placental MSCs could be effectively used for both autologous and allogenic transplantations. The rate of differentiation of MSCs is much quicker, efficient and scalable when compared to ES cells. The soft agar assay indicates that isolated placental MSCs do not possess any malignant property. Several animal as well as human trials have indicated that use of MSCs unlike ES cells does not lead to the formation of teratomas *in vivo *[24]. In addition, usage of term placental MSCs has fewer ethical concerns since they are isolated from foetal tissues that anyway would have been discarded.

## 5. Conclusion

The human term-placenta is relatively easily available and attracts less ethical concerns. Placental tissue constitutes a robust source of MSC. In this study, we investigated several parameters, namely, (1) chromosome number, (2) pluripotency associated gene expression, (3) maternal origin, (4) sequential enzymatic digestion (trypsin followed by collagenase) as methods of isolation, (5) cell propagation, cell cycle, and apoptosis pattern, that are important for their principal utility for cell-based therapy and could influence their proliferative, as well as differentiation, capacities. Based on the results, we conclude that the abundance of pluripotent cells, rapid proliferation, stable karyotype, plasticity and immunomodulatory property make placental MSCs ideal choice for clinical and tissue engineering applications. Nevertheless, the main drawback of using MSCs is that, a panel of surface markers are required for characterization of isolated MSCs for their homogeneity. Further, unlike the adult MSCs, where significant numbers of human clinical trials are underway, use of placental MSCs in clinical applications is relatively new. Additional studies are required to substantiate the use of placental MSCs in medical applications.

## Supplementary Material

For Immunophenotypic characterization of placenta-derived MSC following antibodies (with their vendors name is available) were used as per manufacture's directions and analyzed in a BD FACS analyzer. For the gene-expression studies, PCR was done using primer sequence provided.
