# Supplementary material for: Long-Term Cultured Human Term Placenta-Derived Mesenchymal Stem Cells of Maternal Origin Displays Plasticity
Source: Stem Cells Int. 2012 Mar 26;2012:174328. doi: 10.1155/2012/174328 (PMC3329664; doi:10.1155/2012/174328)
Supplement: Supplementary file 1 — For Immunophenotypic characterization of placenta-derived MSC following antibodies (with their vendors name is available) were used as per manufacture's directions and analyzed in a BD FACS analyzer. For the gene-expression studies, PCR was done using primer sequence provided. [file 174328.f1.pdf]

Supplementary Table 1: Antibody used for flow cytometry and immunostaining.

Antibodies	Conjugate	Cat No	Company
Anti-Human CD 14	FITC	11-0149	eBioscience
Anti-Human CD 34	FITC	11-0349	eBioscience
Anti-Human CD 45	FITC	11-9459	eBioscience
Anti-Human CD 29	FITC	11-0299	eBioscience
Anti-Human CD 73	FITC	11-0739	eBioscience
Anti-Human CD 90	PE	12-0909	eBioscience
Anti-Human CD 105	APC	17-1057	eBioscience
Mouse IgG1 K isotype control	FITC	11-4714	eBioscience
Mouse IgG1 K isotype control	PE	12-4714	eBioscience
Mouse IgG1 K isotype control	APC	17-4714	eBioscience
Anti-NeuN	-	MAB377	Millipore
Anti-Map2	-	MAB378	Millipore
Anti-Neuro filament	-	MAB1592	Millipore
GFAP	-	MAB	Millipore

Supplementary Table 2: Primers for RT-PCR

HLA-DRα Fwd	ATGGCCATAAGTGGAGTCCCTGTGC;
HLA-DRα Rev	CCTGCGTTCTGCTGCATTGCTTTTGCGCACTCC
HLA-DRβ1 Fwd	GTCATTTCTTCAATGGGACGGAGCG;
HLA-DRβ1 Rev	CGCCGCTGCACTGTGAAGCTCTC
Recoverin Fwd	GGAAAAGCGAGCCGAGAAGA
Recoverin Rev	CCTGGGGTGGATGTGTGTGT
Calbindin2 Fwd	GCGGCTACATTGACGAGCAT
Calbindin2 Rev	GCAGCAGAAGCAGGGTTTGG
Pancreatic Amilase Fwd	AGTTAGCTATAAATTATGCACAAG
Pancreatic Amilase Rev	TGCTTGGAAGCATCAAGTCTGAAC

Supplementary Table 3: Primers for Q PCR

B actin F	CCT TCC TGG GCA TGG AGT CCT
B actin R	GGA GCA ATG ATC TTG ATC TTC
Endo Oct4 F	CCT CAC TTC ACT GCA CTG TA
Endo Oct4 R	CAG GTT TTC TTT CCC TAG CT
Endo Sox2 F	CCC AGC AGA CTT CAC ATG T
Endo Sox2 R	CCT CCC ATT TCC CTC GTT TT
Nanog F	GCT TGC CTT GCT TTG AAG CA
Nanog R	TTC TTG ACT GGG ACC TTG TC
